# Deep self-supervised machine learning algorithms with a novel feature elimination and selection approaches for blood test-based multi-dimensional health risks classification

**DOI:** 10.1186/s12859-024-05729-2

**Published:** 2024-03-08

**Authors:** Onder Tutsoy, Gizem Gul Koç

**Affiliations:** Adana Alparslan Turkes Science and Technology University, Adana, Turkey

**Keywords:** Blood test, Deep machine learning, Feature elimination, Feature selection, Health risks classification, Self-feature weighting, Self-supervised learning

## Abstract

**Background:**

Blood test is extensively performed for screening, diagnoses and surveillance purposes. Although it is possible to automatically evaluate the raw blood test data with the advanced deep self-supervised machine learning approaches, it has not been profoundly investigated and implemented yet.

**Results:**

This paper proposes deep machine learning algorithms with multi-dimensional adaptive feature elimination, self-feature weighting and novel feature selection approaches. To classify the health risks based on the processed data with the deep layers, four machine learning algorithms having various properties from being utterly model free to gradient driven are modified.

**Conclusions:**

The results show that the proposed deep machine learning algorithms can remove the unnecessary features, assign self-importance weights, selects their most informative ones and classify the health risks automatically from the worst-case low to worst-case high values.

## Introduction

Blood carries crucial elements including the oxygen, carbon dioxide, nutrients and waste materials to whole body and also protects humans against various infections [1]. Over the centuries, the blood test is performed for a variety of purposes such as the preventive health, early diagnose and treatment observation. It is the most common healthcare tool used in almost every step of the medical processes and every day millions of blood tests are carried out. Even though they are easy to perform and cost effective, due to growing and aging population, spreading health services to large populations and necessitating long treatments; the number of blood test demands increase persistently. This causes an unbearable burden on the economies of the countries since managing such large number of blood tests daily require a great number of medical staff and equipment [2]. In addition, errors because of the staff taking blood samples, technician examining in the laboratories and doctors making decisions are common and unavoidable. To ease and eliminate such problems, developing digital technologies with the advanced data mining and machine learning algorithms are the most feasible and reasonable options. Therefore, this paper proposes a deep self-supervised machine learning algorithm enriched with a feature elimination, self-feature weighting and novel feature selection approaches for the blood test-based multi-dimensional health risk classification.

To gain insights from the blood test results without consulting a health professional, a number of approaches including the simple statistical, feature elimination, feature selection, machine learning and recently deep learning approaches are considered. In terms of the statistical approaches, Krouwer et al. stated that the medical errors in the blood glucose monitoring can likely cause patient harm and the direct statistical approaches including the error grids, Bland–Altman and mountain plots are able to reveal the allowable glucose error [3]. Skirzhytski et al. proposed the discriminant statistical analyses to gain information about the pre-processed semen and blood mixtures data collected by Raman spectra [4]. They also performed an automatic mapping of the samples and cross-validation approaches to validate the statistical results. Song et al. investigated the link between the blood levels of 25 (OH)D and type 2 diabetes risk by applying meta-analysis, DerSimonian-Laird’s random effect model and quadratic spline regression statistical approach [5]. Their research confirmed that the spline regression model identifies that the higher 25 (OH)D levels associate with a lower diabetes risk. Latz et al. focused on understanding the connection between the ABO blood type and significance of the COVID-19 from being slightly infected to intubated by implementing the univariate analyses and logistic regression [6]. The research outcome was that the patients with the blood type B and AB were prone to test positive whereas the blood type O was unlikely to test positive. Although the statistical approaches are useful tools to gain insights from the blood tests, they can usually deal with a single dimensional data and do not form a prediction model.

With respect to the feature elimination approaches, they are generally developed and implemented to discard ubiquities and normal data. For the best of the authors’ knowledge, they have not been yet considered for the blood test-based analyses, but widely implemented in various medical problems. Alshanbari et al. enriched the weighted radial kernel support vector machine with a recursive feature elimination algorithm to estimate and classify the COVID-19 admissions into intensive care units [7]. Similarly, Yu et al. designed a risk prediction model based on a backward feature elimination algorithm to estimate acute on chronic liver failure in hepatitis B patients with severe exacerbation [8]. The algorithm requires a variety of test results including the blood test and aims to identify the patients at high risks early. Proitsi et al. examined the association of the blood lipids and Alzheimer disease with random forest and bootstrap feature elimination algorithms [9]. They performed univariate and multivariate analyses and exhibited associations between the six blood lipids with brain atrophy. All these feature elimination approaches require labelling of the data and consider a single variable. However, raw blood test data is unlabelled and also multi-dimensional. Thus, it requires multi-dimensional self-supervised learning formulation.

Together with the feature elimination approaches, feature selection approaches are developed to pick the most informative data for learning. Shankar et al. considered optimal feature selection based multi-kernel support vector machine algorithm for thyroid disease classification [10]. The resulting algorithm yielded improvement in the classification accuracy and computational efficiency through selecting the best features with the grey wolf optimization algorithm. Berminghan et al. investigated the genomic of a man with the unsupervised and supervised feature selection approaches including the ranking of trait specific genome wide association, pruning based on median distance and re-arranging conditional value approaches [11]. These feature selection approaches combined with the Bayesian model to estimate the height, high density lipoprotein cholesterol (HDC) and body mass index (BMI) of the Croatian and British individuals. Wang et al. utilized meta-heuristic random forest unsupervised feature selection approach for the segmentation of the retinal blood vessels which is substantially important for diagnosing diseases particularly the diabetic retinopathy, hypertension and cardiovascular [12]. Their results confirmed that automatic learning from the raw data and predicting the unknown patterns by selecting the most crucial features is achievable. All these feature selection approaches prioritize whether the data is informative or not. But one or more blood test data can have the values from the worst-case low to worst-case high, while the rest are normal. Therefore, high-dimensional labelling-based feature selection approaches should be developed for the blood test data.

In regard to the machine learning approaches applied to the blood test data, Aktar et al. investigated the severity of COVID-19 patients with the decision tree, random forest, variants of gradient boosting machine and *k-*nearest neighbour machine learning algorithms [13]. The results implied that a number of clinical parameters, particularly the blood samples, can discriminate the positive patients and also label the stage of the diseases. Similarly, Brinati et al. implemented extremely randomized trees, logistic regression and Naïve Bayes machine learning approaches trained with the white blood cells and the platelets to predict COVID-19 patients [14]. The research showed that the machine learning can be an alternative tool to rRT-PCR test kits for detecting the pandemic patients. To model blood glucose dynamics which poses challenges in accurate prediction due to poor diet and time entry information, Woldaregay et al. applied recurrent neural network, feed forward neural network, self-organizing maps, Gaussian process and genetic algorithms [15]. The resulting model was capable to produce locally valid predictions under the certain circumstances, but generated poor global predictions. Even though these machine learning algorithms can receive multiple inputs and generate multiple predictions, they are usually sensitive to the input data, computationally costly and also their decision process is time consuming.

With relation to the deep learning approaches, Rehman et al. modelled the Acute Lymphoblastic Leukemia, that can be detected from the blood test and bone marrow images, with the deep convolutional neural networks and compared the results with the traditional Naïve Bayesian, *k-*nearest neighbours and support vector machines algorithms [16]. The prediction accuracy of the model with the training data was high, but the testing and validation of the model were not carried out. Similarly, Doan et al. examined the stored red blood cells, which are usually assessed through time consuming and complex microscopes, with the convolutional neural networks having an additional feature extraction layer [17]. It is highlighted that the research findings could help to automate complex protocols, reduce laboratory sample handling and minimize procedural errors. Jin et al. proposed a multi-task deep learning network consisting of a feature extraction and tumour segmentation and a classification layer which can perform an instant tumour next state prediction [18]. It is expressed that the resulting deep learning model can capture the tumour dynamics from the longitudinal images and can be utilized for the treatment process of the cancer. Even though these deep learning approaches can handle the large amount of data efficiently, they assume that the successive data is closely dependent. However, this is not a valid assumption for the blood test data since each blood test value carries different information. Henceforth, new and novel deep learning approaches should be developed for the automatic analyses of the blood test data.

Based on the critical review of the recent and related literature above, the key contributions of the paper can be summarized as:

- Designs a multi-dimensional adaptive feature elimination approach to remove the redundant blood test data.
- Forms a self-feature weighting approach to label the data from worst-case low to worst-case high at adaptive intervals.
- Develops a novel multi-dimensional feature selection approach to pick the most informative blood test data.
- Modifies four machine learning algorithms having multiple inputs and multiple outputs to classify the health risks based on the raw blood test data.

In the rest of the paper, section “Proposed deep machine learning algorithm” introduces the proposed deep machine learning algorithm, section “Input–output training data description” forms the input and output training data, section “Deep layer approaches” provides the pre-processing, feature elimination, self-feature weighting and feature selection approaches, section “Machine learning algorithms for optimization” presents the modified machine learning algorithms, section “Results” analyses the results, and finally section “Conclusion and future works” summarizes the paper and states the future works.

## Proposed deep machine learning algorithm

**Fig. 1:**
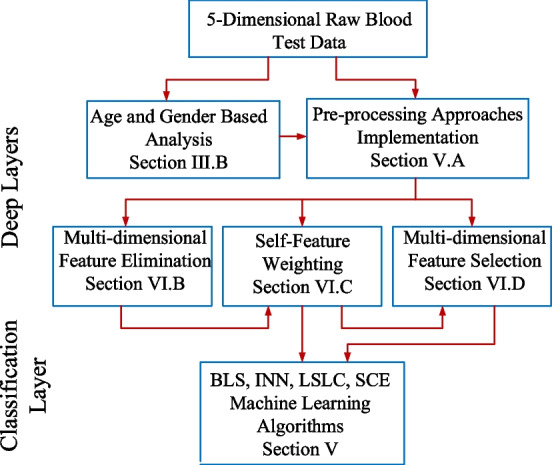
Proposed deep self-supervised machine learning architecture

Figure 1 illustrates the proposed deep self-supervised machine learning algorithm for the blood test-based multi-dimensional health risk classification.

The proposed algorithm initially receives the 5-dimesional raw blood test data and examines with respect to the age and gender to reveal the possible relationship among them. Then simple data pre-processing approaches including the normalization, mean and standard deviations are implemented. Based on these mean and standard deviations, multi-dimensional feature eliminations are performed to remove the normal data which do not carry rich information for the machine learning based optimization problem. Later, features are self-weighted from − 100% to 100%, representing the worst-case low and worst-case high blood test values, respectively. For each interval among the − 100% and 100%, a certain number of features are determined with the multi-dimensional feature selection algorithm. Therefore, with this process, the amount of the data is reduced and the most informative ones are selected for the training and testing of the deep machine learning algorithms. Finally, the constructed data is utilized to optimize the prediction model with the Batch Least Squares (BLS), Iterative Neural Networks (INN), Least Squares with Linear Constraints (LSLC) and Shuffled Complex Evolution (SCE) machine learning algorithms. These 4 algorithms have a variety of properties from reducing the effects of the unknown uncertainties in the data to learning in a constrained space without the gradient information.

## Input–output training data description

Exploring the key properties of the training and testing data greatly contribute to the construction of the input and output data for the machine learning algorithms. This section briefly analyses the data with respect to the gender and age and also groups the multi-dimensional blood test data to process them further. The 5-dimensional blood test data concerned in the paper are hematocrit (HTC), hemoglobin (HGB), white blood cell (WBC), platelet (PLT) and mean platelet volume (MPV). The length of the raw data is 58.490 where 53.459 and 5.031 of them are males and females, respectively. The number of the subjects are 38.595, 13.944, 5.949 and 19.894 in 18–40, 41–50, 51–64 and older than 64 age groups, respectively. The developed feature elimination approach eliminates 32.624 data since they have normal values. The remaining 25.866 are distributed between − %100 and + %100 with the designed feature selection approach.

A. Blood test data analysis in terms of gender

**Fig. 2:**
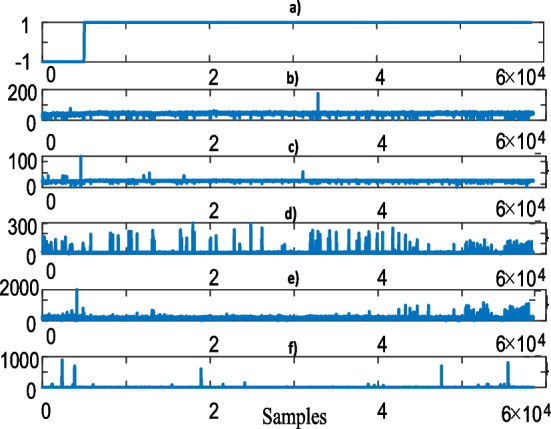
Blood test data with respect to gender, **a** gender where -1 and 1 represent the female and male subjects, **b** HTC, **c** HGB, **d** WBC, **e** PLT, **f** MPV blood test values

Figure 2 visualizes the 5-dimensional blood test data with respect to the gender.

As can be clearly seen from Fig. 2, the number of the male and female subjects are 53.459 and 5.031, respectively. Therefore, the majority of the subjects are male and this hinders us to learn in terms of the gender since it possibly leads to underfitting and overfitting machine learning problems. To reveal the similarities among the blood test data for the female and male subjects, correlation analyses, which essentially identifies the amount of the relationship between two blood test data, can be performed. Let $h^{t}$, $h^{g}$, $w^{b}$, $p^{l}$, $m^{p}$ denote the HTC, HGB, WBC, PLT, MPV blood test data, respectively. Correlation, for example $C_{{h^{t} h^{g} }}$ between $h^{t}$ and $h^{g}$ is simply represented as

1 $$C_{{h^{t} h^{g} }} = \sum\limits_{i = 1}^{n} {\left( {h_{i}^{t} - \overline{h}^{t} } \right)\left( {h_{i}^{g} - \overline{h}^{g} } \right)/\sqrt {\sum\limits_{i = 1}^{n} {\left( {h_{i}^{t} - \overline{h}^{t} } \right)^{2} \left( {h_{i}^{g} - \overline{h}^{g} } \right)^{2} } } }$$

where $\overline{h}^{t}$ and $\overline{h}^{g}$ are the mean values of the $h^{t}$ and $h^{g}$ data, respectively. Table 1 provides the correlation among the whole blood test data.

**Table 1:** Gender-based blood test data correlation. Upper and lower corner values represent the blood test data for the female and male subjects, respectively

Blood test	HTC	HGB	WBC	PLT	MPV
HTC	–	0	0	0	0.88
HGB	0	–	0	0	0.77
WBC	0	0	–	0	0.46
PLT	0	0	0	–	0.08
MPV	0	0	0.11	0	–

As can be seen from Table 1, the female MPV values are correlated with the rest of the blood test data. It is significantly related with the HTC data whereas it has an insignificant relationship with the PLT data. In contrast to the females, the male MPV data is only slightly correlated with the WBC data. Since the number of female subjects is just %8 of the overall subjects, the correlation impact on the machine learning solutions will be ignorable. Next sub-section analyses the blood test data with respect to the age groups.

B. Blood test data analysis in terms of age

**Fig. 3:**
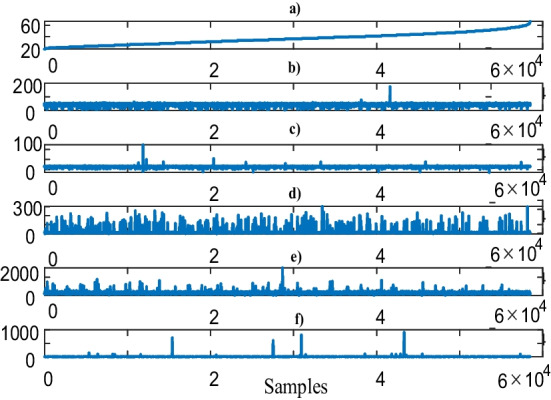
Blood test data with respect to the age groups, **a** age groups, **b** HTC, **c** HGB, **d** WBC, **e** PLT, **f** MPV values

Figure 3 shows the blood test data with respect to the age.

From Fig. 3, it is not straightforward to reveal the relationships among the 5-dimensional blood test data and the age. Therefore, Table 2 is provided to illustrate the correlation between the blood test data divided into two age groups.

**Table 2:** Blood test and age correlation. Upper and lower corner values represent 18–37 and 38–64 age groups, respectively

Blood Test	HTC	HGB	WBC	PLT	MPV
HTC	–	0	0	0	0.98
HGB	0	–	0	0	0.12
WBC	0	0	–	0	0.25
PLT	0	0	0	–	0.30
MPV	0.58	0.44	0.07	0.46	–

The number of subjects in the 18–37 and 38–64 age groups are 32.903 and 25.587, respectively. It is noticeable from Table 2 that the HTC and MPV data are considerably correlated with both age groups. It is also noteworthy that the 38–64 age group MPV data is more firmly related with the rest of the data. This implies that learning the MPV data from the other 4 data is possible. To determine the blood test data-based risks, 5-dimensional data should be grouped for each subject as addressed in next sub-section.

C. Grouping blood test data

**Fig. 4:**
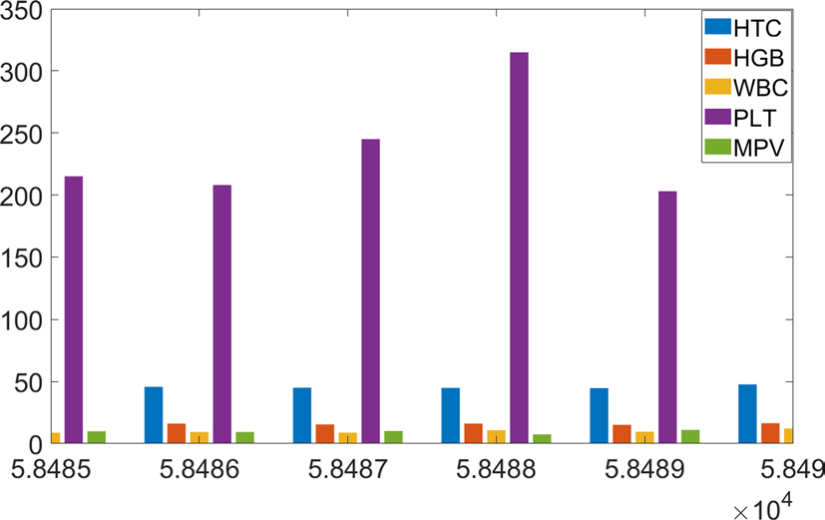
Grouped blood test data for the subjects

Since the machine learning algorithms will make decisions based on the 5-dimensional blood test data, it is important to group them as in Fig. 4 and process them altogether.

It is clear from Fig. 4 that the PLT data is larger than the other 4 data with a varying magnitude. Change in magnitude is a useful property for the learning problems since they carry information about the underlying health problems. However, the larger values always dominate the smaller ones and, in this case, the role of the data with smaller magnitude lessens. Henceforth, the data must be pre-processed and the most informative ones must be chosen as in next section.

## Deep layer approaches

This section initially applies the simple data pre-processing approaches to the blood test data and then introduces the multi-dimensional feature elimination, self-weighting and the novel multi-dimensional feature selection approaches.

A. Pre-processing of the blood test data

**Fig. 5:**
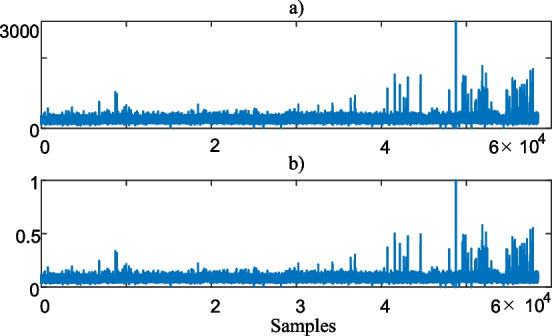
**a** Raw PLT data, **b** normalized PLT data

Since this paper aims to scale the health risks, the blood test data is normalized between 0 and 1. For example, normalized PLT data $p^{ln}$ is

2 $$p_{i}^{ln} = \left( {p_{i}^{l} - p^{{l^{\min } }} } \right)/\left( {p^{{l^{\min } }} - p^{{l^{\max } }} } \right)$$

where $i$ is the data sample index, $p^{{l^{\min } }}$ and $p^{{l^{\max } }}$ are the minimum and maximum values of the $p^{l}$ data, respectively. Figure 5 visualizes the raw $p^{l}$ and normalized $p^{\ln }$ PLT data.

As can be seen from Fig. 5a), the maximum value of the PLT data is around 3000 whereas the maximum value of the normalized PLT data in Fig. 5b) is 1. However, even though the magnitude of the data is normalized, the character of the raw data is maintained as shown in Fig. 5.

Mean and standard deviation of each input data are utilized to eliminate and select the features in Sections B and D. Mean of the normalized PLT data $p_{m}^{\ln } \in {\mathbb{R}}^{N}$, for instance, is given by

3 $$p_{m}^{\ln } = \left( {\sum\limits_{i = 1}^{N} {p_{i}^{\ln } } } \right)/N$$

The corresponding standard deviation $p_{s}^{\ln }$ is expressed as

4 $$p_{s}^{\ln } = \sqrt {\sum\limits_{i = 1}^{N} {\left( {p_{i}^{\ln } - p_{m}^{\ln } } \right)^{2} } } /\sqrt N$$

**Fig. 6:**
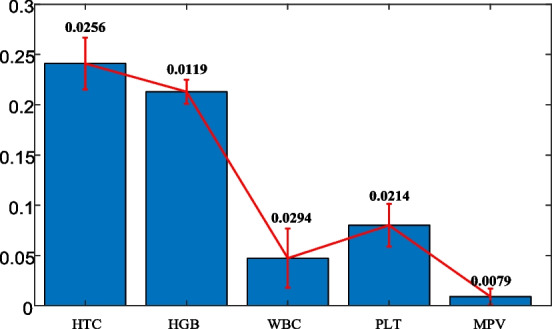
Mean and standard deviations of the blood test data

Figure 6 demonstrates the mean and the standard deviations of the normalized blood test data.

It is clear from Fig. 6 that the HTC and HGB data have larger mean values while the MPV has the smallest one. In addition, the WBC has the largest standard deviation, but the HTC, HGB and PLT have similar standard deviations where the MPV has the smallest. In terms of considering these mean and standard deviation values, next sub-section forms a multi-dimensional adaptive feature elimination approach.

B. Multi-dimensional feature elimination approach

In this paper, a multi-dimensional adaptive feature elimination is performed for three reasons:

- To reduce the dimension of the input–output training and testing data. Therefore, computational time and burden of the algorithms are lessened.
- To develop an adaptive feature elimination approach. Henceforth, the overall data can be manipulated to satisfy the desired machine learning model accuracy.
- To consider 5-dimensional data altogether. In consequence, even one data is close to the abnormality, then the whole data of the subject is utilized.

Algorithm 1 provides pseudocode of the proposed algorithm.

**Algorithm 1:**
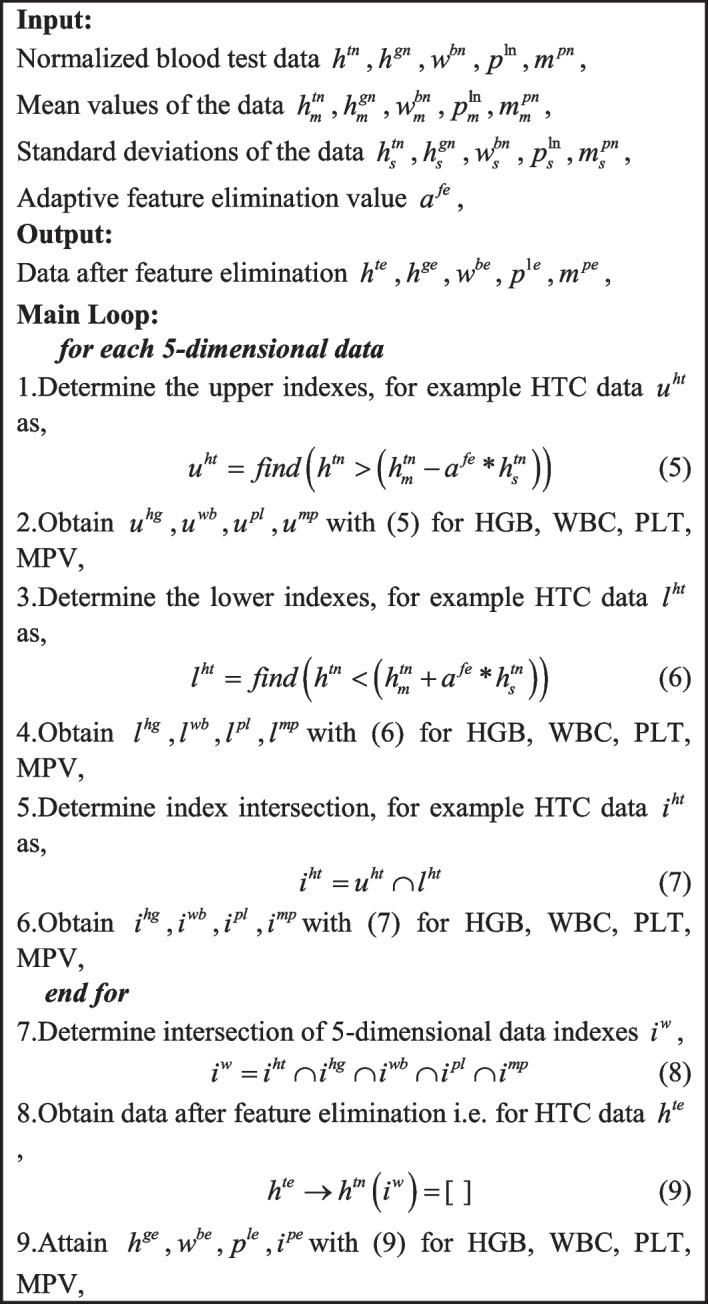
Multi-dimensional feature elimination.

**Fig. 7:**
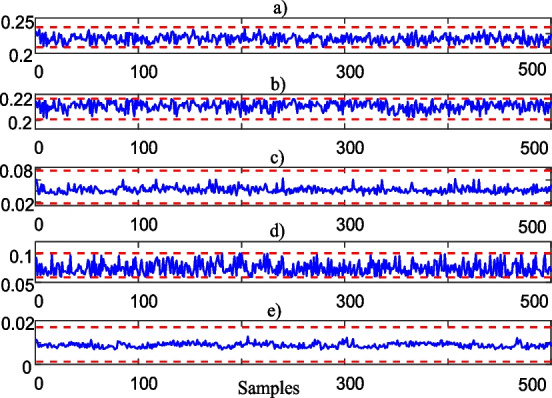
Eliminated features for $a^{fe} = 1$; **a** HTC, **b** HGB, **c** WBC, **d** PLT, **e** MPV. The dashed red lines represent the corresponding upper and lower values to make decisions

Algorithm 1 specifies the eliminated features depending on the adaptive feature elimination value $a^{fe}$ and Fig. 7 shows the eliminated features for $a^{fe} = 1$.

As can be seen from Fig. 7, all the eliminated values are inside the red dashed bounds specified with respect to the mean and standard deviations of each blood test data. It is noticeable that all the eliminated values of the WBC and MPV data are around the centre whereas the rest of the data have values around the upper and lower limits. This occurs because the constructed feature elimination algorithm is 5-dimesional and even one data is outside the boundaries, then the whole data of the subject is kept. Depending on the adaptive feature elimination value $a^{fe}$, the number of the eliminated features vary as illustrated in Table 3.

**Table 3:** The number of eliminated features with $a^{fe}$

$a^{fe}$	0.1	0.5	1	2	3
Eliminated features	28	8.763	32.624	55.155	57.710

As demonstrated by Table 3, the number of the eliminated features increases with respect to the feature elimination value $a^{fe}.$ Therefore, by manipulating this value, it is possible to determine the length of the data that will be utilized for the training and testing. The remaining data is further processed in the self-feature weighting and selection steps introduced next.

C. Self-feature weighting approach

To select the most informative features for learning, Algorithm 2 introduces the self-feature weighting approach.

**Algorithm 2:**
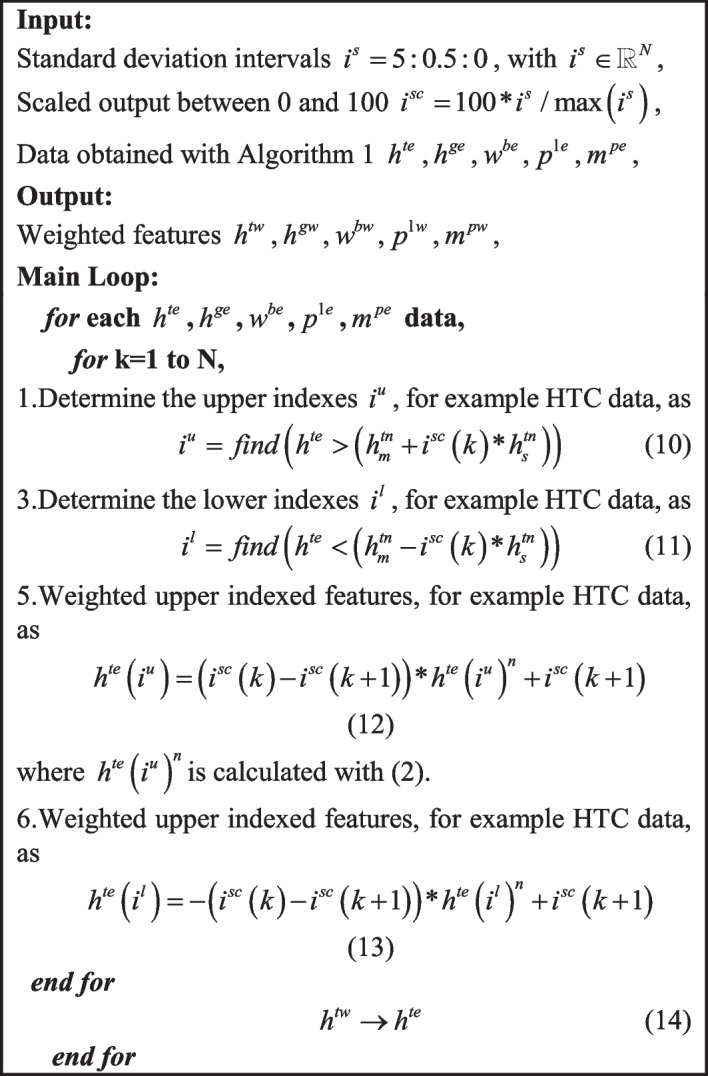
Self-Feature weighting approach.

**Fig. 8:**
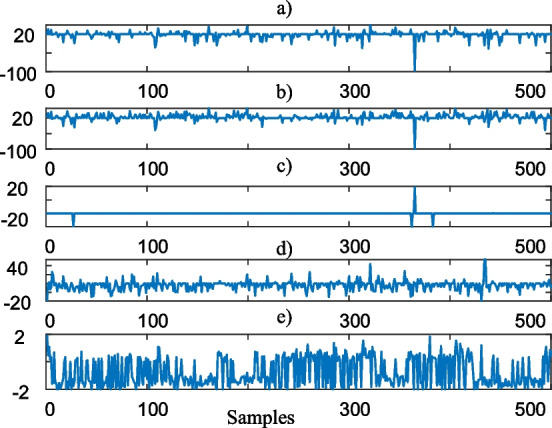
Self-weighted features (partial view); **a** HTC, **b** HGB, **c** WBC, **d** PLT, **e** MPV

As shown in Fig. 8, Algorithm 2 weights the blood test data between -100% and 100% representing the subjects having the lowest and largest abnormal blood test values, respectively.

By considering these self-feature weighted blood test values, next sub-section selects the most informative features.

D. Novel multi-dimensional feature selection approach

Certain number of features should be selected for each label output between − 100% and 100%. Algorithm 3 introduces the multi-dimensional feature selection approach to form the training and testing data.

**Algorithm 3:**
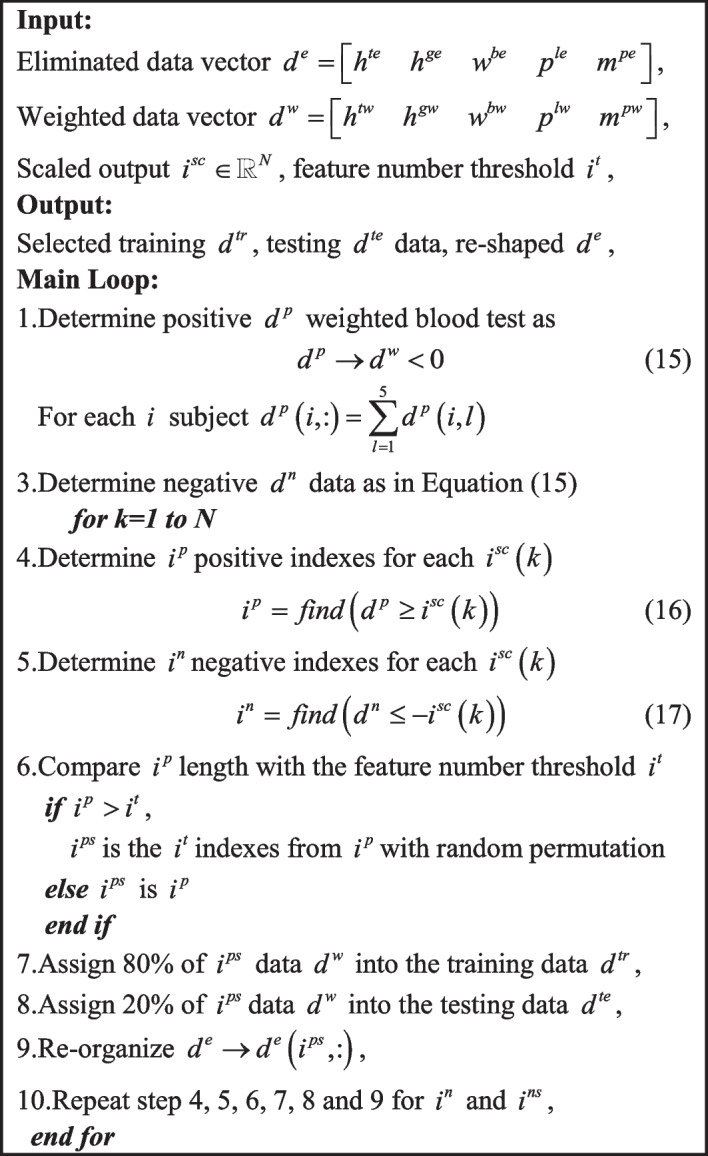
Multi-dimensional feature selection approach.

The Algorithm 3 selects the required amount of informative data as in Table 4.

**Table 4:** Selected features $i^{p}$, $i^{ps}$, $i^{n}$, $i^{ns}$ are existing positive, selected positive, existing negative and selected negative features for each labelling intervals, respectively

Intervals	$i^{p}$	$i^{ps}$	$i^{n}$	$i^{ns}$
± 100%	1	1	1.303	300
± 90%	81	81	16.380	300
± 80%	11	11	4.877	300
± 70%	19	19	2.048	300
± 60%	34	34	604	30
± 50%	60	60	213	213
± 40%	75	75	101	101
± 30%	5	5	35	35
± 20%	0	0	19	19
± 10%	1	1	1	1

Table 4 confirms that the existing $i^{p}$ features are always less than the required 300 features. Thus, the blood test data does not contain sufficient amount of positive weighted data and this deteriorates the performance of the machine learning algorithms. In terms of the existing $i^{n}$ and selected $i^{ns}$ features, they have sufficient negative weighted data up to − 60% and then even though the required 300 features are not provided, the amounts are still large until − 30%. This implies that the machine learning algorithms will perform properly from − 100% to − 30%. This is acceptable since the weighted data between − 30% and − 10% are close to the normal data. Next section provides the machine learning algorithms which optimize the constructed data in this section.

## Machine learning algorithms for optimization

This section initially forms the prediction model structure and then reviews the BLS, INN, LSLC and SCE machine learning algorithms.

A. Prediction model structure

The prediction model $\hat{y}$ is constructed as

18 $$\hat{y} = w^{T} b$$

where $w$ is the unknown parameter vector and $b$ is the basis function formulated as

19 $$b = \left[ {\begin{array}{*{20}c} {1^{N \times 1} } & {\left\| {d^{e} } \right\|^{1/2} } & {\left\| {d^{e} } \right\|^{1} } & {\left\| {d^{e} } \right\|^{1.2} } & {\left\| {d^{e} } \right\|^{2} } & {\left\| {d^{e} } \right\|^{3} } & {\left\| {d^{e} } \right\|^{4} } \\ \end{array} } \right]$$

where $d^{e} \in {\mathbb{R}}^{N \times 5}$ is the re-organized training data vector in Algorithm 3. One can summarize the properties of the basis function in Eq. (19) as

- It carries crucial information processed through the feature elimination in Algorithm 2 and feature selection in Algorithm 4. Thus, it does not consist of raw data.
- It covers a bias, linear and higher order data with partial fractions. This allows to formulate a certain parameter space rather than randomly constructed one.
- It is comprehensive since it has a 7-dimensional parameter space.
- Its dimension is $b \in {\mathbb{R}}^{N \times 35}$ and all of them are utilized for instant learning. Henceforth, uncertainties stemmed from the unknown sources are lessened.

The real output is

20 $$y = d^{tr}$$

where $d^{tr}$ is self-weighted features with Algorithm 2 and selected with Algorithm 3. Next sub-section reviews the BLS algorithm which optimizes the unknown parameters $w$ in Eq. (18).

B. Batch least squares (BLS) algorithm

To derive the BLS parameter learning rule for $w$ in Eq. (18), consider the labelled real output $y$ in Eq. (20) and the prediction model output $\hat{y}$ in Eq. (18). The prediction error $e$ is

21 $$e = y - \hat{y}$$

To generate a smooth unknown parameter space, square the prediction error $e$ in Eq. (21) and manipulate as

22 $$\begin{aligned} e^{2} &= \left( {y - w^{T} b} \right)^{T} \left( {y - w^{T} b} \right) \\ &= y^{T} y - wb^{T} y - y^{T} w^{T} b + wb^{T} w^{T} b \\ \end{aligned}$$

Gradient of the quadratic prediction error in Eq. (22) with respect to the unknown parameters $w$ is given by

23 $$\frac{{\partial e^{2} }}{\partial w} = - 2b^{T} y + 2b^{T} bw$$

Applying the stationary condition on Eq. (23) gives the unknown parameter vector $w$ learning rule expressed as

24 $$w = \left( {b^{T} b} \right)^{ - 1} b^{T} y$$

The BLS update rule in Eq. (24) utilizes whole data to learn the unknown parameters and this is a useful property for the data with large variances. However, to learn the time-varying character of the data, INN algorithm reviewed next is advantageous.

C. Iterative neural network (INN) algorithm

The INN algorithm updates the unknown parameters $w$ in Eq. (18) by iteratively optimizing the squared error in Eq. (21) as

25 $$w_{k + 1} = w_{k} - \frac{\eta }{2}\frac{{\partial e_{k}^{2} }}{{\partial e_{k} }}\frac{{\partial e_{k} }}{{\partial w_{kk} }}$$

where $\eta$ is the learning rate, $k$ is the iteration index. Solving partial derivatives in Eq. (25) yields

26 $$w_{k + 1} = w_{k} - \eta e_{k} b_{k}$$

Both the BLS and INN introduced in sub-sections B and C are unconstrained optimization algorithms which can assign larger importance to some parameters than the others. Thus, next sub-section formulates the LSLC constrained mathematical optimization algorithm.

D. Least squares with linear constraints (LSLC) algorithm

The LSLC algorithm focuses on minimization of the prediction error with the equality and inequality parameter constraints $\alpha$ and their upper value $p$ defined as

27 $$\begin{aligned} & \mathop {{\text{min}}}\limits_{{w_{k}^{*} }} \quad \frac{1}{2}\left\| {y_{k}^{{}} - \hat{y}_{k}^{{}} } \right\|_{2} \\ & {\text{subject to}}\;\;\;\;\left\| {w_{k}^{{}} } \right\|_{2} \le p\left\| \alpha \right\|_{2} \\ & \quad \quad \quad \quad \quad \alpha < w_{k}^{{}} < p\alpha \\ \end{aligned}$$

The constrained optimization problem in Eq. (27) can be expressed with Lagrange multipliers given by

28 $$L\left( {w_{k}^{{}} ,\lambda } \right) = \frac{1}{2}\left\| {y_{k}^{{}} - w_{k}^{{^{T} }} b_{k}^{{}} } \right\|_{2}^{2} + \frac{\lambda }{2}\left( {\left\| {w_{k}^{{}} } \right\|_{2}^{2} - p^{2} \left\| \alpha \right\|_{2}^{2} } \right)$$

Obtaining derivative of $L\left( {w_{k}^{{}} ,\lambda } \right)$ with respect to the $w_{k}^{{}}$ is

29 $$\left( {b_{k}^{{^{T} }} b_{k}^{{}} + \lambda } \right)w_{k}^{{}} = b_{k}^{{^{T} }} y_{k}^{{}}$$

Determining derivative of $L\left( {w_{k}^{{}} ,\lambda } \right)$ with respect to the $\lambda$ is

30 $$\left\| {w_{k}^{{}} } \right\|_{2}^{2} - p^{2} \left\| \alpha \right\|_{2}^{2} = 0$$

Re-organizing (29) as $w_{k}^{{}}$ is on the left and substituting in Eq. (30) yields

31 $$\left\| {\frac{{b_{k}^{{}} y_{k}^{{}} }}{{b_{k}^{{^{T} }} b_{k}^{{}} + \lambda }}} \right\|_{2}^{2} - p^{2} \left\| \alpha \right\|_{2}^{2} = 0$$

Solving (31) for $\lambda$ and substituting (29) leads to the LSLC parameter update rule given by

32 $$w_{k + 1} = w_{k} + \eta^{c} \frac{{b_{k}^{T} }}{{b_{k}^{T} b_{k} + \lambda }}y_{k}$$

where $\eta^{c}$ is the LSLC learning rate. Next sub-section reviews the SCE machine learning algorithm.

E. Shuffled complex evolution (SCE) algorithm

SCE in Algorithm 4 is a model and gradient free meta-heuristic algorithm which essentially searches for the optimal solutions in a pre-defined parameter space.

**Algorithm 4:**
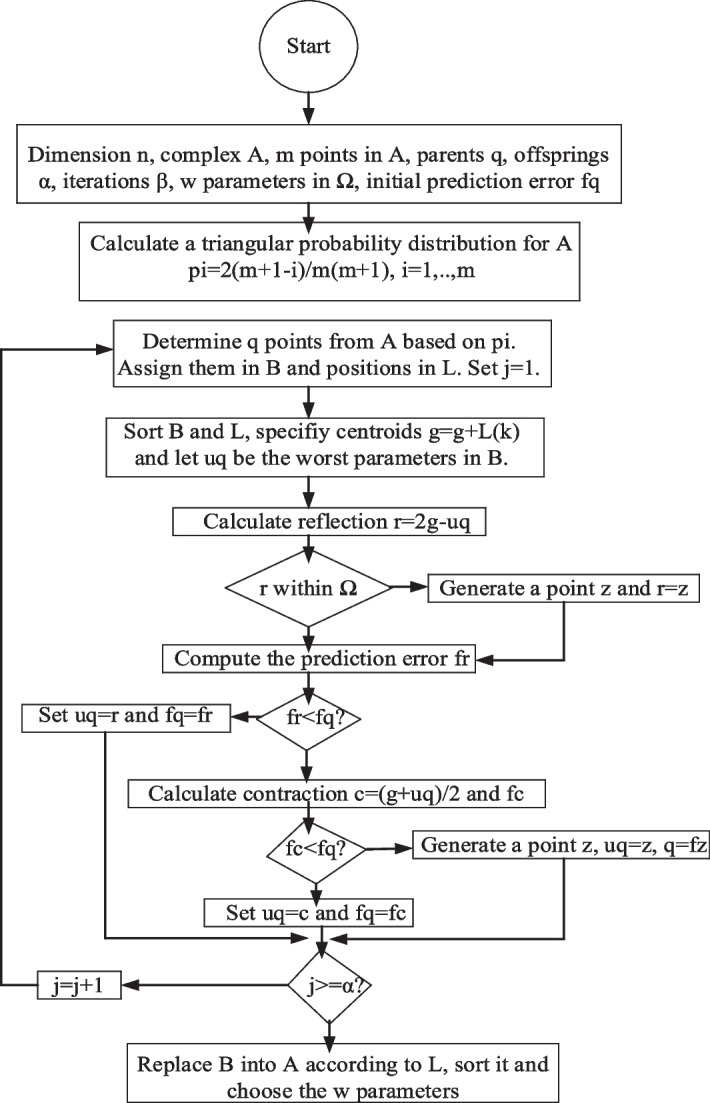
The SCE machine learning algorithm.

The next section analyses the results extensively.

## Results

This section initially presents the parameters of the machine learning algorithms and then analyses the results extensively.

A. Parameters of the machine learning algorithms

Next sub-section analyses the training results obtained with the machine learning parameters in Table 5.

**Table 5:** Parameters of the machine learning algorithms

Parameters	Value	Description
$n$	10	Algorithm 4
$A$	4	Algorithm 4
$q$	5	Algorithm 4
$\alpha$	3	Algorithm 4
$\beta$	5	Algorithm 4
$\Omega$	100	Algorithm 4
$f_{q}$	0	Algorithm 4
$\eta = \eta^{c}$	0.05	Equation (26)
$i^{t}$	300	Algorithm 2
$a^{fe}$	1	Algorithm 1

B. Machine learning results with training data 

**Fig. 9:**
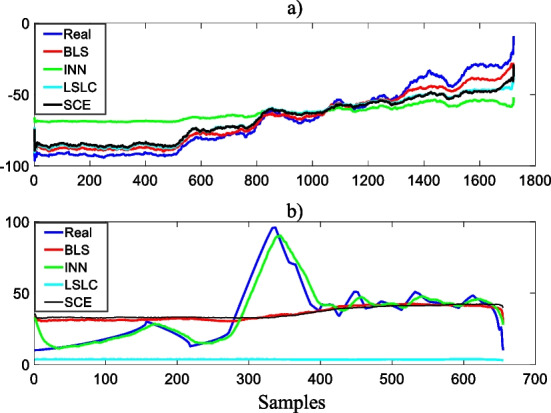
Learning with training data; **a** low, **b** high values

Figure 9 shows the results with the constructed training data.

As can be seen from Fig. 9a, the BLS, LSLC, SCE algorithms can capture the overall character of the abnormally low blood test data whereas the INN algorithm fails to learn the labelled output around − 100%. This is due to iterative nature of the INN, which forgets the previous learning as the sample horizon approaches 0% values. It is also noticeable that the BLS algorithm closely follows the real output since it has ability to reduce the effects of the unknow uncertainties. With respect to Fig. 9b, due to insufficient and imbalanced abnormally high blood test data discussed with the context of Table 4, all the machine learning algorithms except the INN cannot manage to learn the desired output. However, because of the large variance in learning, its robustness will be poor. To clearly express the efficiencies of the machine learning algorithms, next sub-section provides statistical analyses of the developed models.

C. Statistical analyses of the deep learning algorithms

**Fig. 10:**
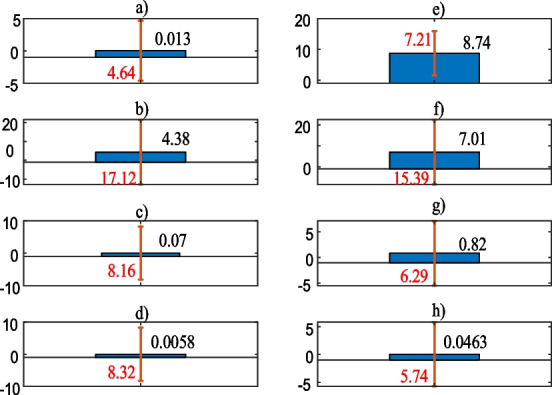
Mean error **a**–**d** are the training results, **e**–**h** are the testing results of the BLS, INN, LSLC, SCE algorithms, respectively

Figure 10 compares the predictions of the deep self-supervised machine learning algorithms with the training and testing data.

As can be seen from Fig. 10a and e, the BLS algorithm has considerably large mean error with the test data since it is prone to the variations due to it matrix inversion requirement. With respect to the INN algorithm in Fig. 10b and f, it yields almost similar mean errors, but nearly two times larger standard deviations with the test data. In terms of the LSLC algorithm in Fig. 10c and g, it has 0.07 mean error with the training data and it jumps to 0.82 with the test data. However, the test data with the LSLC leads to smaller standard deviation than the training data. With regard to the SCE algorithm in Fig. 10d and h, it produces the smallest mean error both with the training and testing data and similar to the LSLC algorithm, it generates lower standard deviation with the test data. Next section summarizes the paper and expresses the future direction of the research.

## Discussions

As addressed in section “Deep layer approaches” B, multi-dimensional raw blood test data can be separated as the normal and abnormal. The 5-dimensional raw blood test data is labelled as abnormal in the presence of at least one abnormal test value. This will enable us to make more advanced health risk decisions and will allows us to map these results to the possible underlying illnesses. The abnormal multi-dimensional blood test data is labelled automatically without requiring an expert knowledge in Section “Deep layer approaches” C. The labelling classes are adaptive; henceforth, they can be adjusted immediately in case of emergencies or changing conditions. The labelled abnormal data is assigned into the classes with the feature selection approach in Section “Deep layer approaches” D. Since this algorithm is capable of selecting sufficient amount of data, conventional overfitting and underfitting machine learning problems can be avoided. In order to determine the most appropriate machine learning algorithms which can capture the unknown patterns of the selected multi-dimensional blood test data, four machine learning algorithms are implemented and it is shown that the INN is unable to learn the desired classes due to its time-varying nature.

## Conclusion and future works

This paper proposed a deep self-supervised machine learning algorithm enriched with the multi-dimensional adaptive feature elimination, self-weighting and novel feature selection approaches to automatically learn from 5-dimensional raw blood test data. The initial analyses of the data with the basic statistical approaches showed that there is no significant correlation between the gender and 5-dimensional blood test data (Table 1). In addition, it is proved that the MPV data is highly correlated with the HTC data only for the 18–37 age group (Table 2). Adaptive property and reliability of the feature elimination approach were illustrated (Table 3). Finally, a novel multi-dimensional feature selection approach was formed and the selected features presented (Table 4). The research outcomes showed that all the algorithms were able to capture the worst-case low blood test values where the LSLC algorithm yielded the largest estimation bias (Fig. 9). However, for the worst-case high values, only the INN algorithm managed to learn the dynamics of the corresponding blood test values due to its iterative nature.

The main disadvantages of the research are that the number of the female subjects are much less than the male subjects, diversity of the abnormal data is quite limited, the amount of the worst-case positive data is insufficient compare to the worst-case negative ones. In addition, multi-layer machine learning algorithms should be examined in addition to the four machine learning algorithms considered in this paper. A further drawback of the study is that the dataset does not have any missing data, but in real-life applications it is possible that the blood test data can have randomly distributed missing data.

As a future work, the proposed deep machine learning algorithms should be expanded by including the treatment policies that can manipulate the future responses of the blood test data.

## Data Availability

The datasets used during this research are available from the corresponding author O.T., e-mail otutsoy@atu.edu.tr on request. Declarations
